# Expression of genome defence protein members in proliferating and quiescent rat male germ cells and the *Nuage* dynamics

**DOI:** 10.1371/journal.pone.0217941

**Published:** 2019-06-10

**Authors:** Letícia Rocha-da-Silva, Lucia Armelin-Correa, Isabelle Hernandez Cantão, Verena Julia Flaiz Flister, Marina Nunes, Taiza Stumpp

**Affiliations:** 1 Laboratory of Developmental Biology, Department of Morphology and Genetics, Escola Paulista de Medicina, Universidade Federal de São Paulo (EPM/UNIFESP), São Paulo, Brazil; 2 Department of Biological Sciences, Universidade Federal de São Paulo (UNIFESP), Diadema, Brazil; Massachusetts General Hospital, UNITED STATES

## Abstract

During epigenetic reprogramming germ cells activate alternative mechanisms to maintain the repression retrotransposons. This mechanism involves the recruitment of genome defence proteins such as MAEL, PIWIL4 and TDRD9, which associate with piRNAs and promote Line-1 silencing. MAEL, PIWIL4 and TDRD9 form the piP-bodies, which organization and dynamics vary according to the stage of germ cell epigenetic reprogramming. Although these data have been well documented in mice, it is not known how this mechanism operates in the rat. Thus, the aim of this study was to describe the distribution and interaction of MAEL, PIWIL4, TDRD9 and DAZL during rat germ cell development and check whether specific localization of these proteins is related to the distribution of Line-1 aggregates. Rat embryo gonads at 15 days post-conception (dpc), 16dpc and 19dpc were submitted to MAEL, PIWIL4, TDRD9 and DAZL immunolabelling. The gonads of 19dpc embryos were submitted to the double-labelling of MAEL/DAZL, TDRD9/MAEL and PIWIL4/MAEL. The 19dpc gonads were submitted to co-immunoprecipitation assays and fluorescent *in situ* hybridization for Line-1 detection. MAEL and TDRD9 showed very similar localization at all ages, whereas DAZL and PIWIL4 showed specific distribution, with PIWIL4 showing shuttling from the nucleus to the cytoplasm by the end epigenetic reprogramming. In quiescent 19dpc gonocytes all proteins colocalized in a *nuage* adjacent to the nucleus. DAZL interacts with PIWIL4 and MAEL, suggesting that DAZL acts with these proteins to repress Line-1. TDRD9, however, does not interact with DAZL or MAEL despite their colocalization. Line-1 aggregates were detected predominantly in the nuclear periphery, although did not show homogeneous distribution as observed for the *nuage*. In conclusion, the *nuage* in quiescent rat gonocytes show a very distinguished organization that might be related to the organization of Line-1 clusters and describe the association of DAZL with proteins responsible for Line-1 repression.

## Introduction

It has been exhaustively described that the male germ cells undergo very special mechanisms during their pre-natal development. One of these mechanisms is epigenetic reprogramming, when they undergo bulk DNA hypomethylation and *de novo* methylation. The other one is quiescence, which takes place just after epigenetic reprogramming and comprehend the end of pre-natal period and the beginning of the post-natal life. During the hypomethylation period of the epigenetic reprogramming, these cells activate alternative mechanisms to maintain the silencing of DNA methylation-regulated retrotransposons [1] [2]. This alternative mechanism involves the activation of genome defence genes such as *Mvh (Ddx4)*, *Piwi* family, *Tex19*.*1*, *Mov10l1*, *Mael* and *Tdrd* family, which expression starts to be detected as the genome becomes hypomethylated [3] [4] [5] [6]. The proteins corresponding to these genes are then recruited and, along with piRNAs, promote retrotransposon silencing through DNA methylation or mRNA degradation. *Dazl*, an important gene with germ cell-specific expression, has also been suggested to play a role in the genome defence against retrotransposon expression, since it regulates the translation of *Mvh* [7] and *Tex19*.*1* [8]. However, the direct participation of DAZL in this mechanism has not been directly investigated.

Retrotransposons are transposable elements that use mRNA intermediates to move themselves throughout the genome. They constitute around 40% of the whole mammalian genome and most of them are no longer able to transpose [9] [10] [11]. However, the Line-1 family of retrotransposons, which comprises around 17% to 20% of human genome, include some active members. The activity of these Line-1 retrotransposons must be strictly controlled to avoid harmful insertions in the genome that could lead to gene mutation. As previously mentioned, this control requires the assemble of genome defence proteins and piRNA that act in both nuclear and cytoplasmic cell compartments. The piRNAs used in the control of Line-1 expression are produced from Line-1 sequences (or clusters) in a poorly understood mechanism named ping pong biogenesis [4] [12] in which the proteins PIWI, MVH, MAEL and TDRD are indispensable [13] [14] [15] [5].

The assembly of the protein/piRNA complexes that promote retrotransposon silencing and piRNA biogenesis lead to the formation of germ cell-specific structures called *nuage*. The *nuage* are dynamic non-membranous, electron-dense structures present in the cytoplasm of germ cells [16]. Morphological studies described the presence of the nuage as the intermitochondrial cement or as the chromatoid body detected in specific phases of germ cell development [16] [17] [18]. It is now clear that the *nuage* are RNA processing centres that contain different protein associations, and that they change their distribution and/or localization according to the phase of germ cell development and differentiation [4] [19] [20]. An example is MAEL shuttling between nucleus and cytoplasm during drosophila oogenesis [21]. Alterations in MAEL distribution and localization was also observed in mice. In 14.5dpc male gonads, MAEL is localized throughout the germ cell cytoplasm, whereas at 16.5dpc it starts to accumulate close to the nucleus and is also detected inside the nucleus [4]. Although the functional relationship between these differences and the phase of germ cell development is not known, the changes in MAEL localization described in mice occur between the end of the proliferative phase (14.5dpc) and the quiescent period (16.5dpc). The quiescence period of the male germ cells (that can also be called gonocyte) can be considered very particular since it is not related to terminal differentiation or senescence but instead precedes the formation of stem cells (the spermatogonial stem cells) that express some pluripotency markers [22]. Very little is known about the quiescent gonocyte, but it has been suggested that this phase might represent a cell cycle checkpoint, since this is a moment when they do not die or proliferate at all [23].

Despite the fact that exquisite studies about genome defence mechanisms developed by germ cells have been performed and important data has been provided, how these mechanisms operate is still obscure. Even less is known about these mechanisms during the germ cell quiescence, especially in the rat. Previous study by our group showed that some aspects of rat germ cell development are different from mice. Thus, the aim of this study was to look at the expression of genes and proteins involved in the male germ cell genome defence during the transition from proliferating to quiescent phase in order to identify possible differences between these two moments of germ cell embryonic development. We show that the interaction and distribution of the proteins DAZL, MAEL, TDRD9 and PIWIL4 differ from rat and mice and that they present a very distinguishing localization in quiescent gonocytes.

## Methods

### Animals and collection of embryonic and postnatal gonads

This study was carried out according to the rules of National Institutes of Health (NIH) guide for the care and use of Laboratory animals and was approved by the ethics committee for animal use from the Federal University of Sao Paulo (6075040914).

Male rats and embryos (*Rattus norvegicus albinus*) were obtained from time-mated females provided by the Center for Development of Experimental Models for Medicine and Biology (CEDEME) at Paulista Medicine School (EPM)—Federal University of Sao Paulo (UNIFESP). The pregnancy was confirmed by vaginal smears taken at 7 a.m. in the morning after the matings. The day when sperm were detected in the smear was considered 1 day post-conception (dpc). The animals were kept in plastic cages under a 12/12 hour light/dark cycle at 23–25°C. Food and water were allowed *ad libitum*. The dams were submitted to euthanasia using the method of anaesthesia/analgesia (xylazine/ketamine, 10 mg/Kg and 100mg/Kg, respectively) and euthanized by cardiac incision. The embryos were collected at 15, 16 and 19dpc. Adult (70dpp) testes were also collected. The adult rats were euthanized using the anaesthesia/analgesia previously described. Thymus of the adult rats was also collected and used as controls for the Western blot analysis, as described later.

### Immunohistochemistry and immunofluorescence

To describe the *nuage* of the rat germ cells, the embryos (15 and 16dpc, n = 6) and testes (19dpc, n = 6) were fixed in Bouin’s solution and processed for paraffin embedding. From each embryo or testis four 6μm-thick sections were obtained and submitted to immunolabelling as follows. The sections were dewaxed in xylene, hydrated and submitted to heat antigen retrieval using citrate buffer (pH 6.0) for 10 minutes. The slides were treated with 5% BSA for 30 min and incubated with the primary antibodies anti-DAZL (1:100; Abd-Serotec MCA2336), anti-MAEL (1:100; Abcam, ab106713), anti-PIWI4 (1:200; Abcam, ab87939), and anti-TDRD9 (1:100; Abcam, ab118427). For immunohistochemistry, the slides were washed in PBS (0.05 M, pH 7.2) 3x and incubated with the secondary antibody (DAKO Detection System—K0690, USA). The slides were washed again in PBS and then incubated with the streptavidin-peroxidase (DAKO Detection System—K0690, USA). The reaction was revealed with DAB (K3468, DAKO, USA) and the nuclei were stained with Harris Hematoxilyn. For MAEL/DAZL, TDRD/DAZL, and PIWIL4/DAZL double-labelling, FITC anti-rabbit (Abcam, Ab6791), Alexa anti-mouse (Invitrogen, A10036) and Texas Red-conjugated secondary antibodies were used. DAPI was used for nuclear staining. Negative controls (primary antibody omission) were performed for all reactions.

Because anti-MAEL, anti-PIWIL4 and anti-TDRD9 antibodies were produced in rabbit, the double labelling could not be performed using antibodies. Then, anti-DAZL, which was produced in mouse, was used for the co-localization assays, allowing to infer about the co-localization of the other target proteins.

The slides were carefully analysed and the pattern of protein detection in germ cells was described and documented using the Leica Image Analysis System LAS (Cambridge, England) for immunohystochemistry and the NIS Element (Nikon) for Immunofluorescence.

### Co-immunoprecipitation (Co-IP) and Western blot

With the aim to investigate the interaction of the proteins that compose the rat germ cell *nuage*, a co-immunoprecipitation assay was performed at 19dpc. This age was chosen based on the data obtained by the immunolocalization assays. Thirty testes from 15 embryos obtained from 5 different mothers were collected and sonicated in lysis buffer containing protease inhibitor (cOmplete, Roche—Cat.: 04693132001). Co-IP was performed using the Co-Immunoprecipitation Pierce Kit (Cat.: 26149), according to the manufacturer’s instructions. The extract of the gonads was immediately directed to protein quantification by the BradFord method, with a BSA standard curve, using the Bio-rad protein assay kit (Biorad, #5000002). Part of the sample was incubated overnight at 4°C in the spin columns baited with anti-DAZL or anti-TDRD9 antibodies. After incubation, the columns were centrifuged (1000xg, 1 min) and washed with specific buffers for precipitation of the proteins (prey) that may have interacted with the bait antibody coupled to the column (elution product). The bait proteins (DAZL or TDRD9) were not removed from the columns in the wash protocol used here (see the instructions provided by the manufacturer for details). The elution product (expected to contain the prey proteins), the post-Co-IP extract (expected to have few or no prey and bait proteins) and the remaining whole extract were saved for the Western blotting analysis, as described below. This experiment was carried out three times (n = 3).

### Western blotting

The sample obtained by Co-IP, as well as part of the total testis extract, were incubated with Laemlli buffer (125 mM Tris-HCl, 4% SDS, 8% Glycerol, 0.004% bromophenol blue and 10% mercaptoethanol) in a dry bath at 100°C for 5 min and running gel (SDS-PAGE). The proteins were transferred to PVDF membranes previously activated with methanol. The membranes were incubated with blocking buffer (PBS; 0.1% Tween-20; 5% milk) at 37°C and then with the antibodies anti-DAZL (1:400), anti-MAEL (1:400), anti-PIWI4 (1:200) or anti-TDRD9 (1:300) for 16h. β-Actin (1:100; Cell Signalling, #4970) was used as endogenous control. Then, the membranes were washed 3x with PBS containing 0,1% Tween 20 and incubated with the secondary antibodies anti-mouse (1:15000, G021040 Life Technologies) or anti-rabbit (1:10000, G21234 Life Technologies) for 1h. The membranes were analysed using the Luminata Forte kit (Merck Millipore, Cat. WBLUF0100).

### Fluorescent *In-Situ* Hybridization (FISH)

DNA extraction from adult rat testis was performed according to [18] and submitted to PCR using degenerate primers designed for conserved Line-1 regions (L1F: 5′- CCATGCTCATSGATTGG-3′ e L1R: 5′-ATTCTRTTCCATTGGTCTA-3′), according to [24]. The amplicon was precipitated by incubation with 2μl t-RNA (Thermo AM7119), 1/10vol of sodium acetate (3M pH 5.2) and 2vol of cold 100% ethanol, overnight, at -20°C. It was then centrifuged at 14000xg, for 30mim at 4°C, washed with 70% ethanol and resuspended in ultrapure water. The amplicon was submitted to Dig-Nick Translation (Roche, Cat. No 11 745 824 910) for 7 min at 15°C. The reaction was blocked by incubating the sample with 0,5 M EDTA (pH 8.0) at 65°C for 10 min. Then labelled sequences were precipitated as previously described and stored at -20°C.

The testes of 19dpc embryos (n = 3) were fixed in Carnoy’s solution for paraffin embedding. The slides were dewaxed in xylene, hydrated and incubated with 0.2N HCl solution for 20min at room temperature (RT). The sections were then incubated proteinase K for 20 min- RT (5μg/ml, 25mMTris-HCl, 5mM EDTA). The slides were washed with PBS and incubated with formamide solution, 2x sodium citrate (SSC, pH 7.0) overnight at 4°C. The DNA and Line-1 probes were denaturated at 75°C for 20min and 5min, respectively. The sections were, then, dehydrated in ethanol and hybridized with the Line-1 probes for three days. After this period, the sections were washed with specific buffers (2x SSC and 50% formamide and Tween20) at 42°C. The reaction was blocked with 3% BSA solution at RT and the sections were incubated with anti-digoxigenin-rhodamine (Roche Cat# 11207750910, 1:200). Finally, the slides were sealed with Vectashield (Vector) and observed in a fluorescence microscope using the image analysis system NIS Element (Nikon). Negative controls were performed by omitting the incubation with the Line-1 probe (S1 Fig).

## Results

### PIWL4, DAZL, MAEL and TDRD9 labelling in germ cells

**Fig 1:**
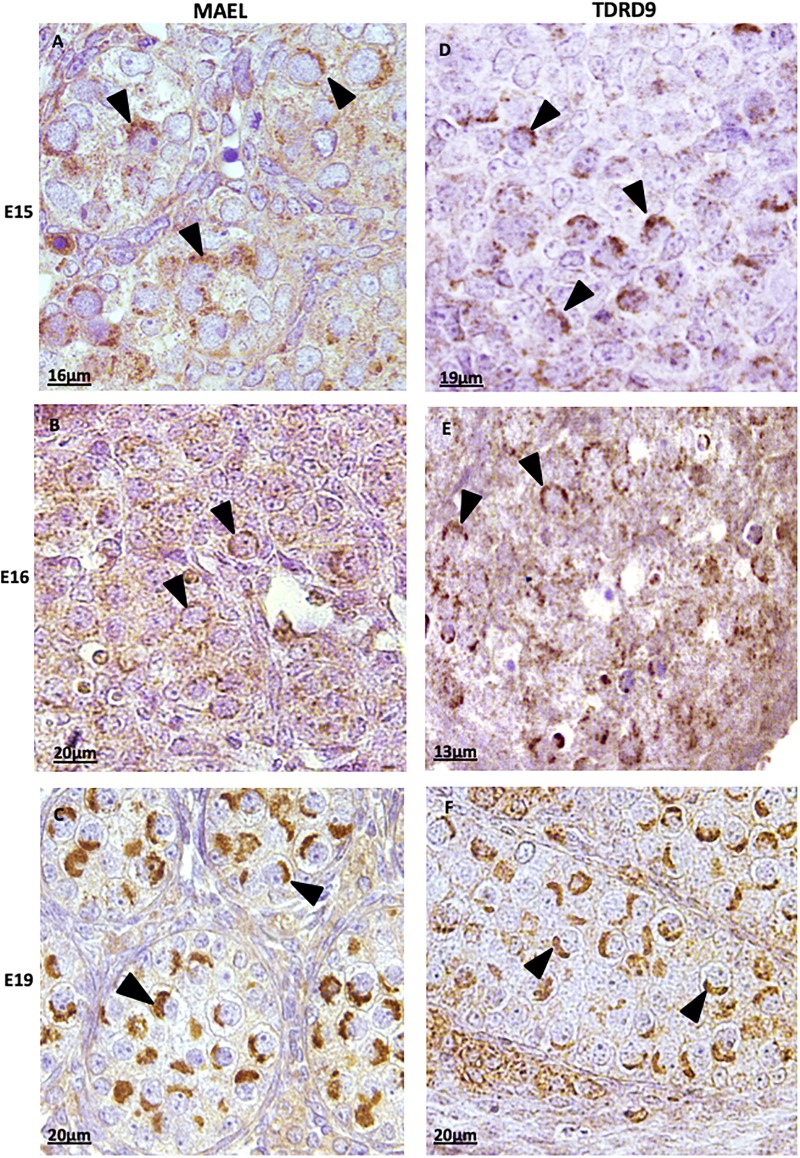
Immunodetection of MAEL (A, B and C) and TDRD9 (D, E and F) in rat embryo gonads. At 15dpc **(A** e **D)** and 16dpc **(B** e **E)** the *nuage* (arrowheads) are distributed more homogeneously around the gonocyte nucleus. At 19dpc **(C** and **F)** the *nuage* (arrowheads) is adjacent to a restricted portion of the gonocyte nucleus.

The detection of MAEL (Fig 1A to 1C) and TDRD9 (Fig 1D to 1F) was very similar. At 15dpc (Fig 1A and 1D) and 16dpc (Fig 1B and 1E) these proteins were detected in the cytoplasm of germ cells showing a granulated pattern close to the nucleus. At 19dpc, however, MAEL (Fig 1C) and TDRD9 (Fig 1F) were detected in a restricted region of the cytoplasm very close to the nucleus.

**Fig 2:**
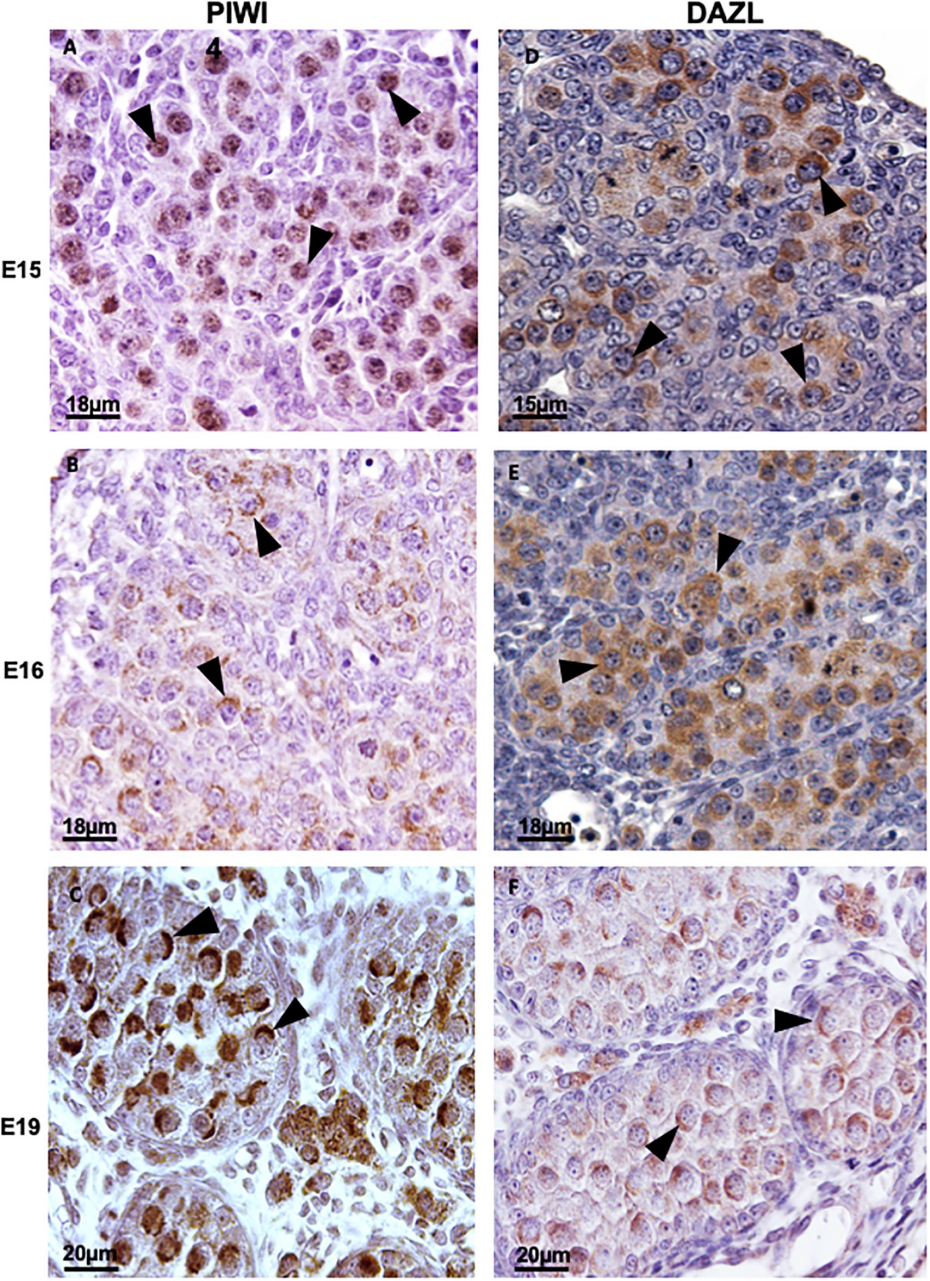
Immunodetection of PIWIL4 (A, B and C) and DAZL (D, E e F) in rat embryo gonads. At 15dpc **(A)** is present in the nucleus of the (arrowheads), whereas DAZL **(D)** is localized in the cytoplasm, (arrowheads). At 16dpc **(B** and **E)** both proteins are localized in the cytoplasm (arrowheads), but PIWIL4 distribution is more restricted to the region close to the nucleus (arrowheads), whereas DAZL is diffusely distributed throughout the cytoplasm (arrowhead). At 19dpc **(C** and **F)**, as observed for MAEL and TDRD9, the *nuage* (arrowheads) is is adjacent to a restricted portion of the gonocyte nucleus.

PIWIL4, however, showed different localization at 15dpc and 16dpc, changing its location from the nucleus to the cytoplasm. At 15dpc PIWIL4 was detected in the nucleus of germ cells (Fig 2A). At 16dpc this protein was detected in germ cell cytoplasm and was distributed in granules of different sizes (Fig 2B). At 19dpc PIWIL4 was detected in the cytoplasm but was restricted to a region very close to the nucleus (Fig 2C), as described for TDRD9 and MAEL.

DAZL labelling at 15dpc (Fig 2D) and 16dpc (Fig 2E) was present throughout the whole germ cell cytoplasm and, differently from PIWIL4, MAEL and TDRD9, was not distributed in distinguishable granules. On the other hand, at 19dpc (Fig 2F) DAZL showed exactly the same labelling pattern observed for the other proteins investigated here, i.e., it was restricted to a region very close to the nucleus.

**Fig 3:**
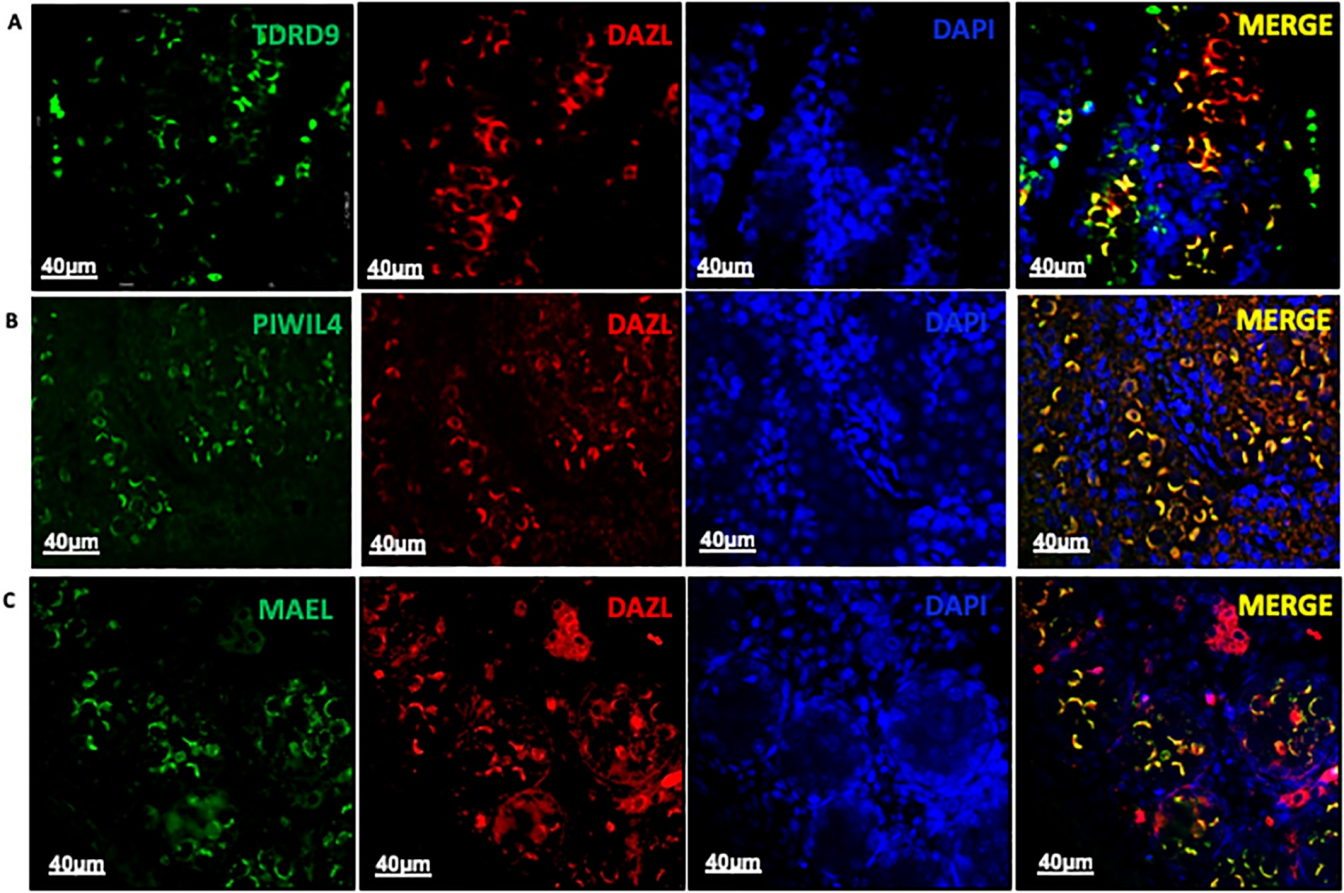
Colocalization of DAZL/TDRD9 (A), DAZL/PIWIL4 (B) and DAZL/MAEL (C) in the 19dpc gonads. The colocalization can be observed by the yellow/orange color in merge.

All four proteins analyzed here formed a cap in the nucleus at 19dpc (Figs 1C, 1F, 2C and 2F) that seem to cover nearly half of it. Because of this similarity of the labelling pattern, we performed a double immunofluorescence labelling to check whether these proteins indeed co-localize with each other. Because good quality anti-MAEL, anti-PIWIL4 and anti-TDRD9 antibodies available for assays using the rat were all raised in rabbit and only DAZL was produced in mouse, we performed the immunolabelling of DAZL/MAEL, DAZL/TDRD9 and DAZL/PIWIL4. Thus, if DAZL co-localized with these three proteins, we would be able to suggest that these proteins co-localize with each other. Indeed, DAZL localization coincided with TDRD9 (Fig 3A), PIWIL4 (Fig 3B) and MAEL (Fig 3C) in the 19dpc testis, suggesting that these four proteins show the same localization in the rat testes at 19dpc.

### Analysis of DAZL and TDRD9 Interactions by Co-IP Assay

**Fig 4:**
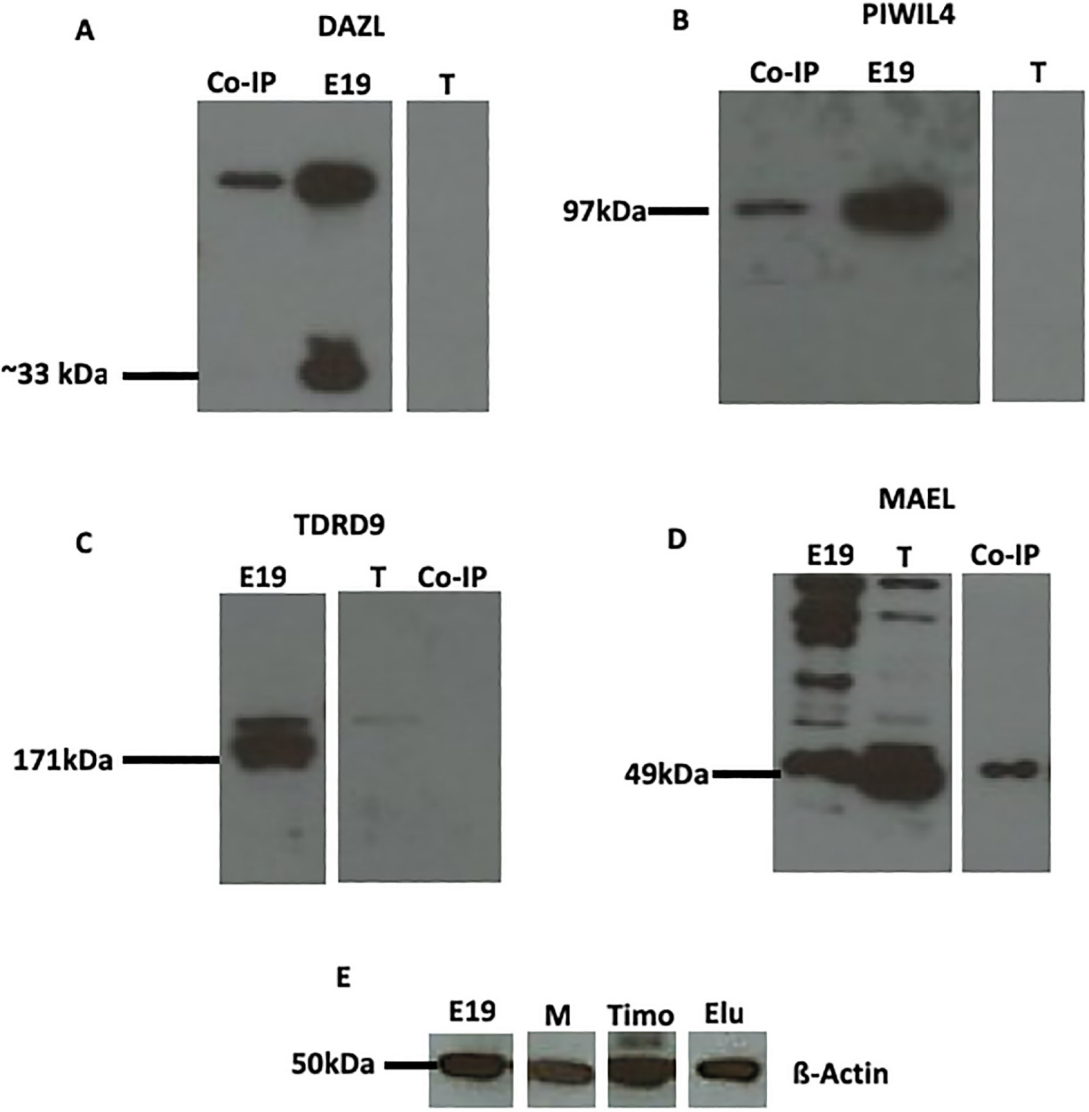
Western Blotting for MAEL, DAZL, PIWI4 and TDRD9. **A)** DAZL, that has been used as the bait, was present in the whole gonad extract (E19) and is absent from the Co-IP yield (Co-IP). **B)** PIWIL4 was detected in the Co-IP yield (Co-IP) and in the whole gonads extract (E19). **C)** TDRD9 was detected in the whole extract (E19), but not in the Co-IP yield (Co-IP). **D)** MAEL, as observed for PIWIL4, was detected in the whole extract and in the Co-IP yield (Co-IP). **E)** ß-Actin was used as the reference protein. T: testis; M: muscle.

**Fig 5:**
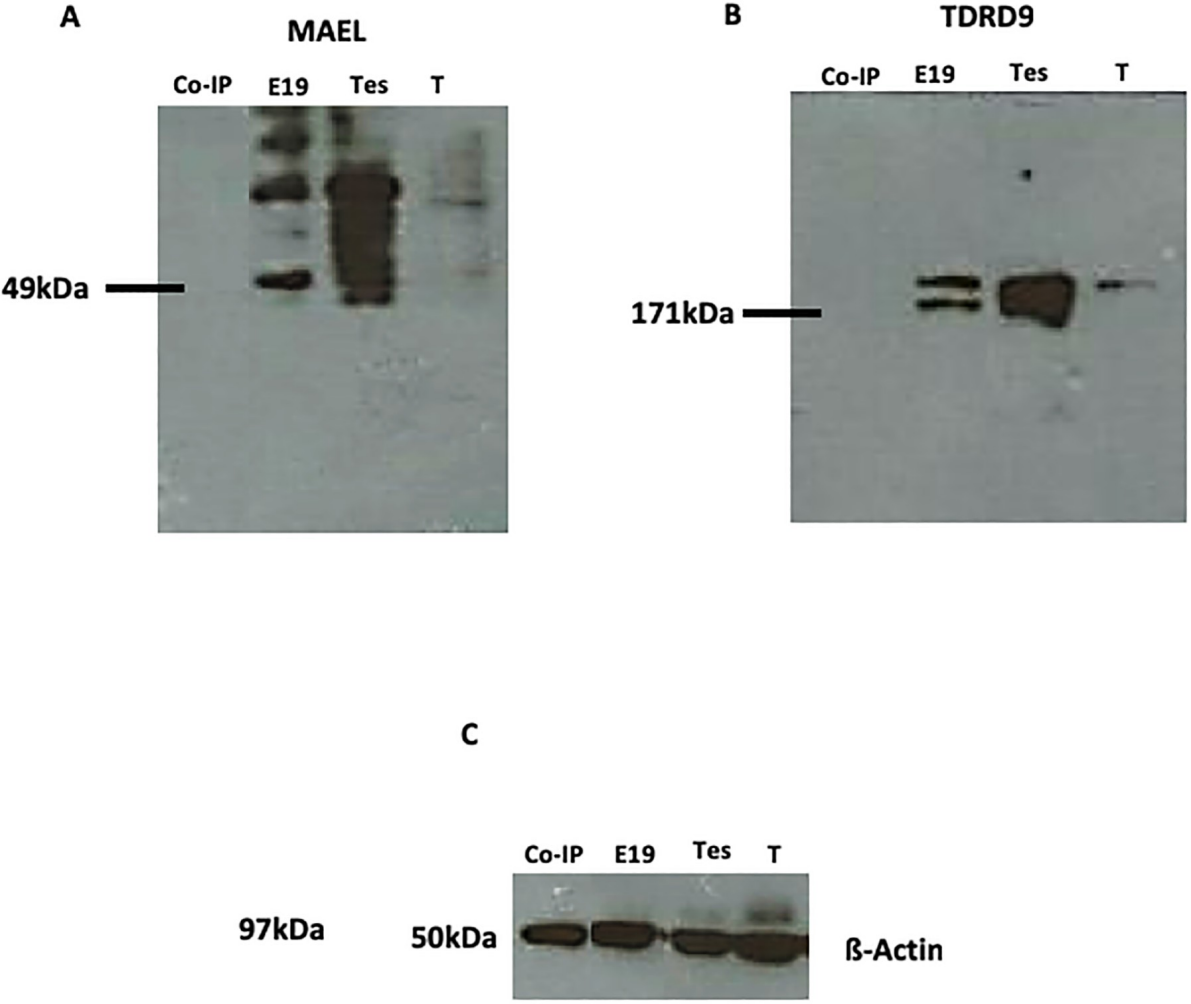
Western Blotting for MAEL and TDRD9. **A)** MAEL was detected in the whole extract (E19) and in the testis (Tes), but not in the Co-IP yield (Co-IP). **B)** As observed for MAEL, TDRD9 was detected in the whole extract (E19) and in the testis (T), but not in the Co-IP yield (Co-IP). **C)** ß-Actin was used as the reference protein.

In face of the DAZL/MAEL, DAZL/TDRD9 and DAZL/PIWIL4 co-localization results previously described, we performed a coimmunoprecipitation assay using DAZL or TDRD9 as baits to confirm whether DAZL interacts with MAEL, TDRD9 and PIWIL4 and whether TDRD9 interacts with MAEL. The Western blot analysis of the immunoprecipitated yield using DAZL as bait showed a band after incubation with anti-MAEL and anti-PIWIL4 antibodies, but not after incubation with anti-MAEL antibody (Fig 4), indicating that DAZL interacts with MAEL and PIWIL4, but not with TDRD9. When TDRD9 was used as the bait protein, the Co-IP yield did show the presence of MAEL (Fig 5), indicating that TDRD9 does not interact with MAEL.

The whole testis extract, as well as post Co-IP extract were also included in the Western blotting. We observed that DAZL, PIWIL4, MAEL and TDRD9 were present in the whole extract, but DAZL and TDRD9 were absent from the post Co-IP extract, as well as in the Co-IP elution yield when they were used as bait (Figs 4 and 5). DAZL and TDRD9 absence from the elution yield can be explained by the washing method used in the Co-IP reaction, which did not include alkaline washes necessary to remove the bait protein from the antibody-coated column.

### Localization of Line-1 sequences by FISH

**Fig 6:**
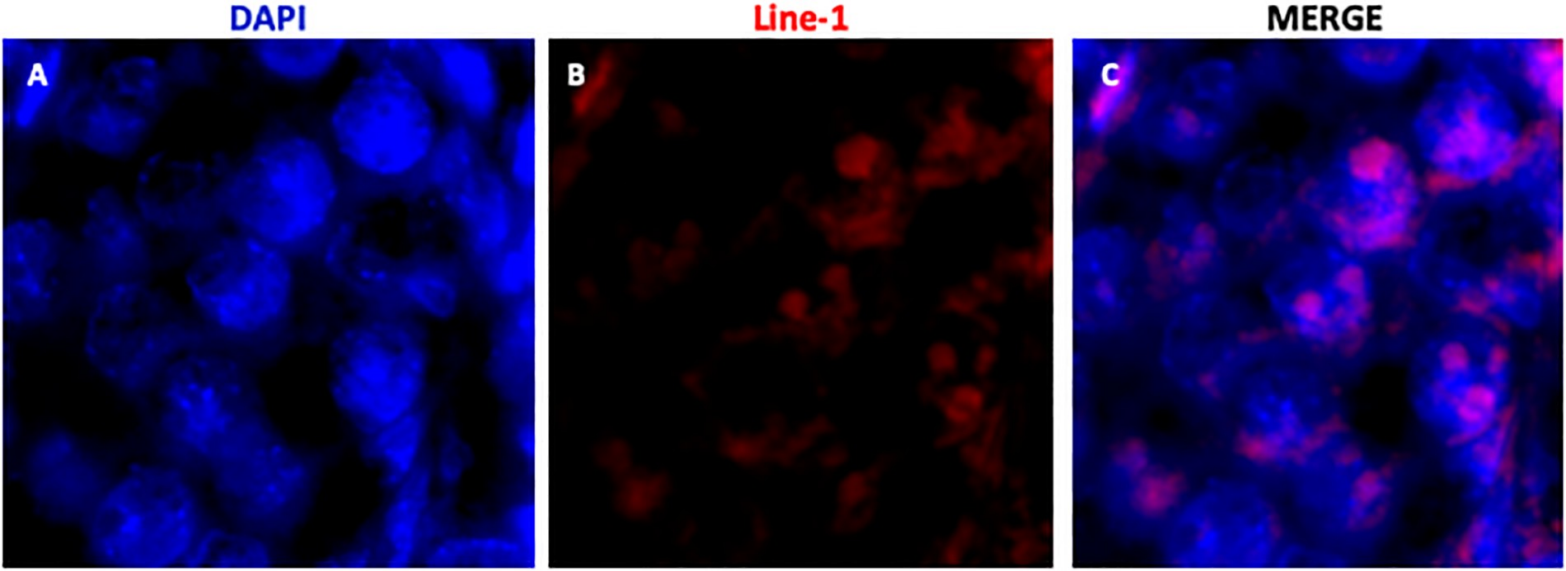
Detection of Line-1 in the nuclear genome by fluorescent *in situ* hybridization (FISH) in the 19dpc gonocytes. Line-1 aggregates are observed more frequently in the periphery of the gonocyte nucleus (arrows). A: DAPI; B: Line-1; C: merge.

To investigate whether the detection pattern of the four proteins analyzed in the 19dpc testes was related to Line-1 distribution in the nuclear genome, we performed a fluorescent *in situ* hybridization (FISH) using rat Line-1 probes. FISH assay showed Line-1-positive regions in the gem cell nuclei and that these regions occupied the nuclear periphery in some germ cells, whereas others showed more central labelling (Fig 6).

## Discussion

The mechanisms of genome defence against the expression of retrotransposons which expression is controlled by DNA methylation is very particular in germ cells, since these cells undergo an intense mechanism of epigenetic reprogramming that could be potentially dangerous for their genome integrity. These genome defence mechanisms include the production of proteins that associate with each other and with piRNAs to constitute cytoplasmic structures called *nuage*. In the *nuage*, piRNAs are processed and guided to promote the silencing of retrotransposons [25]. In a previous study, our group showed that MVH, a protein member of the genome defence machinery, is detected earlier in the rat germ cells than in mouse germ cells [26] Here we show that the *nuage* structure in the rat germ cell is similar to those described in mice, although the distribution of the proteins members of the genome defence machinery as well as their interaction may differ.

Aravin et al. and Shoji et al. showed that, in mouse gonocytes, MIWI2, which correspond to PIWIL4 in rats, forms a subcellular complex with TDRD9 and MAEL [4] [14], what agrees with our results. Interestingly, [4] shows that MIWI2 (our PIWIL4) is detected in the nucleus and in the cytoplasm of 16.5dpc mouse embryos (what correspond to 18–19dpc rat embryos) and become predominantly nuclear by the end of gestational period. Here we observed that PIWIL4 shows the opposite localization, i.e., it was nuclear in male gonocytes at 15dpc and then became cytoplasmatic at 16dpc. The nuclear function of PIWIL4/MIWI2 is related to a direct action on DNA methylation [27] [28]. Thus, this difference in the timing of PIWIL4/MIWI2 detection in the nucleus might be related to the dynamics of epigenetic reprogramming in these cells. In mouse, gonocyte DNA starts to become hypermethylated (*de novo* methylation) at 16.5dpc [29], coinciding with MIWI2 shuttling to the nucleus. In the rat germ cells, we previously suggested that the DNA *de novo* methylation starts at 15dpc [30], when PIWIL4 is still localized in the nucleus. Thus, it seems that PIWL4 nuclear localization in the rat gonocytes coincides with DNA *de novo* methylation as observed for mice.

The detection of MAEL and TDRD9 observed here was exclusively cytoplasmic, contrasting with what has been described for mice, in which these proteins have been detected in both nucleus and cytoplasm [4] [14]. However, it important to mention that these authors described MAEL and TDRD9 during the epigenetic reprogramming period, whereas our analysis started by the end of epigenetic reprogramming. Thus, it is possible that at previous stages these proteins would be detected in the nucleus of the rat gonocytes.

The detection of MAEL, TDRD9, PIWIL4 and DAZL was particularly interesting at 19dpc, when rat gonocytes are quiescent [23]. At this age, all four proteins colocalized in the same region of the gonocyte cytoplasm, arranged in a half moon shape adjacent to the nucleus, despite the differences in their localization at earlier stages. The localization of MAEL, TDRD9 and PIWIL4 in conspicuous *nuage* granules has been described in mice [2] [4] [14]. However, a description of this type of *nuage*, showing so restricted localization, was not found in the literature. It was previously described [4] the RNA MAEL/ TDRD9/PIWIL4-positive processing centres as piP-bodies to discriminate them from the pi-bodies, which contain MILI (PIWIL2) and TDRD1 proteins, and from traditional P-bodies that are not involved in piRNA processing.

It is important to point out that, from all proteins related to germ cell genome defence described in the literature, very little is mentioned about DAZL [31] [32]. On the other hand, our results about DAZL colocalization with these three well defined members of the genome defence and piRNA biogenesis machineries suggest that DAZL might indeed play a relevant role in this mechanism. Based on this finding, we performed a Co-IP assay using DAZL as bait and observed that, although MAEL, TDRD9, PIWIL4 and DAZL colocalize at 19dpc, DAZL interacts with MAEL and PIWIL4 but not with TDRD9. Previous studies showed that, in mouse germ cells, DAZL regulates *Mvh* [7] and *Tex19*.*1* [8] [33], two important genome defence genes, at post-transcriptional level. Thus, since it has been shown that MAEL and PIWIL4 (MIWI2) act to repress Line-1 [13] [28] [34], our results indicate that DAZL might act with PIWL4 and MAEL to repress Line-1 in quiescent rat gonocytes.

TDRD9 is another important protein related to Line-1 repression in mice [14]. Although the interaction between PIWIL4 (MIWI2) and TDRD9 has been described in mouse germ cells [14], our Co-IP data indicates otherwise. This difference can be related to species-specific differences or even to technical issues. In the work by [14] the assay performed to look at TDRD9/MIWI2 interaction was based in the co-transfection of expression vectors to HEK293T cells, whereas we used whole gonad extract.

Line-1 is the most abundant and active retrotransposon in mammals, constituting 17% to 20% of mammalian genome. Considering the importance of MAEL, TDRD9 and PIWIL4 for Line-1 repression, and our data about DAZL interaction with MAEL and PIWIL4, we decided to investigate whether the specific localization of these proteins at 19dpc coincided with Line-1 clusters. Using DNA FISH we show that Line-1 seems to form aggregates in gonocyte nuclei but this nuclear distribution does not show a consistent pattern for all gonocytes as observed for the localization of MAEL, TDRD9, DAZL and PIWIL4 *nuage*. However, the presence of peripheric nuclear labelling for Line-1 could suggest that Line-1 is present in the nuclear periphery to facilitate piRNA delivery to the nuage, what would strenght our hypothesis. Although Line-1 aggregates were not all restricted to one nuclear hemisphere, as observed with the *nuage*, a particular nuclear-nuage interplay during gonocyte quiescence cannot be dismissed, since a relationship between the function and the localization of these proteins have been indicated. Line-1 nuclear distribution was studied by DNA FISH in mouse retina cells [35]. The authors show that Line-1 is localized around the heterochromatin block and in the nuclear periphery in ganglion cell nucleus, and our results show a certain degree of irregular concentration of Line-1 copies in gonocyte nuclear periphery. However, the relationship between this distribution pattern and genome defence mechanisms have not been addressed.

It is important to consider that, although the significance of the quiescence period of quiescence observed in rat and mouse male germ cell remains obscure e, it this period is believed to represent a checkpoint in which germ cells are dedicated to identify and/or correct possible genome damages to then start their differentiation into spermatogonial stem cells. Indeed, testicular germ cell tumours (TGCT), for example, have not been identified in the rat and mouse after the use of potential inductors such as phthalates [36] [37] [38] [39]. It has been shown that LINE-1 is hypomethylated in human TGCT and that this hypomethylation is more pronounced in these tumours than in those of somatic cell origin [40]. Another study shows that piRNA/PIWI machinery is defective in TGCT cells [41]. Thus, considering this data and that gonocyte quiescence might be a checkpoint period, it is possible that the particular localization of the *nuage* observed here is related to a specific mechanism of genome defence necessary during quiescence. Further studies are needed to investigate this issue.

In conclusion, our results also show that DAZL interacts with MAEL and PIWIL4, indicating that DAZL might act with these proteins in repression of the retrotransposon Line-1. We also showed that MAEL, DAZL, PIWIL4 and TDRD9 are present at different stages of rat male germ cell development and that their localization changes as germ cells differentiate, showing a particular *nuage* pattern in quiescent gonocytes. Further studies are necessary to elucidate possible relationship between the *nuage* pattern in quiescent gonocytes and Line-1 repression.

## Supporting information

S1 Fig Negative controls of Line-1 FISH. L1 DNA FISH negative control. All DNA FISH steps were performed but no L1 probe was added to the hybridization buffer. (A) Composite image in gonocytes showing anti-digoxigenin antibody in red (B) and DAPI in blue (C).
(TIF)
