# Supplementary figures and images for: Expression of genome defence protein members in proliferating and quiescent rat male germ cells and the Nuage dynamics
Source: PLoS One. 2019 Jun 10;14(6):e0217941. doi: 10.1371/journal.pone.0217941 (PMC6557511; doi:10.1371/journal.pone.0217941)

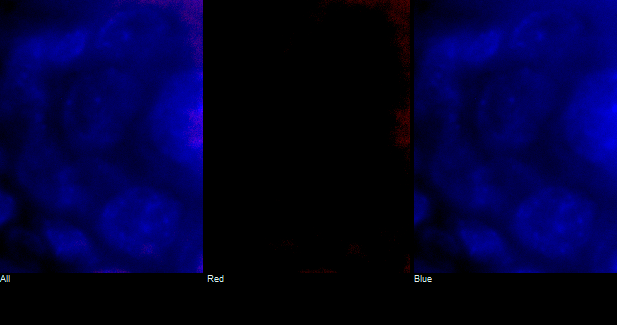

Supplement: S1 Fig — L1 DNA FISH negative control. All DNA FISH steps were performed but no L1 probe was added to the hybridization buffer. (A) Composite image in gonocytes showing anti-digoxigenin antibody in red (B) and DAPI in blue (C). (TIF) [file pone.0217941.s001.tif]
